# Three-Piece Inflatable Penile Prosthesis Implantation for the Treatment of Severe Erectile Dysfunction Following a Complex Pelvic Fracture: A Case Report

**DOI:** 10.7759/cureus.30151

**Published:** 2022-10-10

**Authors:** Minh H Truong, Trung Q Ngo

**Affiliations:** 1 Department of Urology and Renal Transplantation, People's Hospital 115, Ho Chi Minh, VNM; 2 Department of Nephro-Urology and Andrology, Pham Ngoc Thach University of Medicine, Ho Chi Minh, VNM; 3 Department of Urology and Renal transplantation, People's Hospital 115, Ho Chi Minh, VNM

**Keywords:** urethral injury, impotence, pelvic fracture, erectile dysfunction, penile prosthesis implantation

## Abstract

Pelvic fracture is one of the common causes of erectile dysfunction (ED). The pathophysiology of ED following pelvic injury is quite complicated and comprises vascular, neurogenic, corporal, as well as psychogenic causes. Penile prosthesis implantation is the third-line treatment of ED due to any reason including pelvic trauma that poorly responds to other standard treatments. In this study, we reported a case of a 33-year-old man with severe erectile dysfunction and urethral stricture following a complex pelvic fracture due to a traffic accident who was successfully implanted with a three-piece inflatable penile prosthesis at People’s Hospital 115. At the sixth month of follow-up, this device has been working effectively, the patient had the ability to attain full erection for sexual intercourse. Both the patient and the partner are satisfied with their sexual lives. ED is a long-term consequence of pelvic fracture. The high proportion of young patients with a demand for erection rehabilitation and complex pathophysiology make the treatments even more challenging. Penile implant surgery is a potential treatment for refractory ED patients suffering from pelvic trauma.

## Introduction

Pelvic fractures accounted for roughly 3% of all adult bone fractures with complicated management in trauma care. The patients who frequently experience pelvic trauma are young and have high overall injury severity scores (ISS) (25 to 48 ISS), leading to the mortality rates remaining high. High-energy trauma causes bony pelvic ring injuries and concomitantly damages the local soft tissues and organs [1]. Complex pelvic fracture is defined as pelvic ring fractures associated with soft tissue injuries in the pelvic region (urogenital system, rectum, sigmoid, vessel) and with hemodynamic instability of the patient [2]. The bladder and urethra are vulnerable to trauma, which has been reported with a prevalence of 5% to 10%. In addition to severe urological injury, there is also damage to the nerves and blood vessels supplying the genitalia that leads to erectile dysfunction (ED), which is one of the long-term complications [3].

It is anticipated that the overall incidence of sexual dysfunction following pelvic fractures, which ranges between 11% and 30%, is likely to be greater than the previous average of 5% partly due to improved post-trauma survival rates [3]. Notably, this incidence is even higher in patients who have a urethral injury after a pelvic fracture than in those who do not [4]. A combination of neurogenic, vascular, corporal, and psychogenic injury is contributed to the pathophysiology of impotence after the injury of the pelvis [3]. The management of ED patients after pelvic trauma is also similar to the recommended treatments of ED, in general, which includes lifestyle modification, oral phosphodiesterase type 5 inhibitors (PDE5i), penile vacuum devices, intracavernosal injection therapy, and penile implant surgery [5,6]. Therein, penile prosthesis placement should be appropriately considered for patients with refractory ED of any origin, including pelvic injury who have failed or refused more conservative treatments [7]. However, performing this procedure on patients with a pelvic fracture urethral injury (PFUI) has certain risk factors and challenges for experienced surgeons.

Here, we present a case of a 33-year-old man with impotence and urethral stricture after a PFUI he sustained in an accident six years ago. This patient successfully underwent urethral dilation and penile prosthesis implantation at People’s Hospital 115. In this article, we want to share our initial experiences when we performed the surgery.

## Case presentation

A 33-year-old Vietnamese male presented to our hospital with the inability to attain an erection and a weak urinary stream following polytrauma, including a complex pelvic fracture with urethral injury and a femur fracture caused by a truck crash accident that he had six years ago. He had nailing for a right femur fracture and a suprapubic cystostomy for urethral injury. Subsequently, he experienced many procedures of urethral reconstruction, such as end-end anastomosis, urethroplasty with buccal mucosal graft, and now monthly routine urethral dilation, respectively. He lived in Hanoi and had his own business with a normal internal medical profile before this admission. He was referred to our department with the chief complaint of severe ED (impotence) and his International Index of Erectile Function-5 score (IIEF-5) was 5. Also, he had a weak urinary stream when urinating. He had been taking PDE5i medications and using vacuum erectile devices for erection problems for two years, however, he reported the ineffectiveness of these measures. After consulting with andrologists about treatment options, he refused intracavernous injection therapy due to inconvenience and desired penile implant surgery.

**Figure 1:**
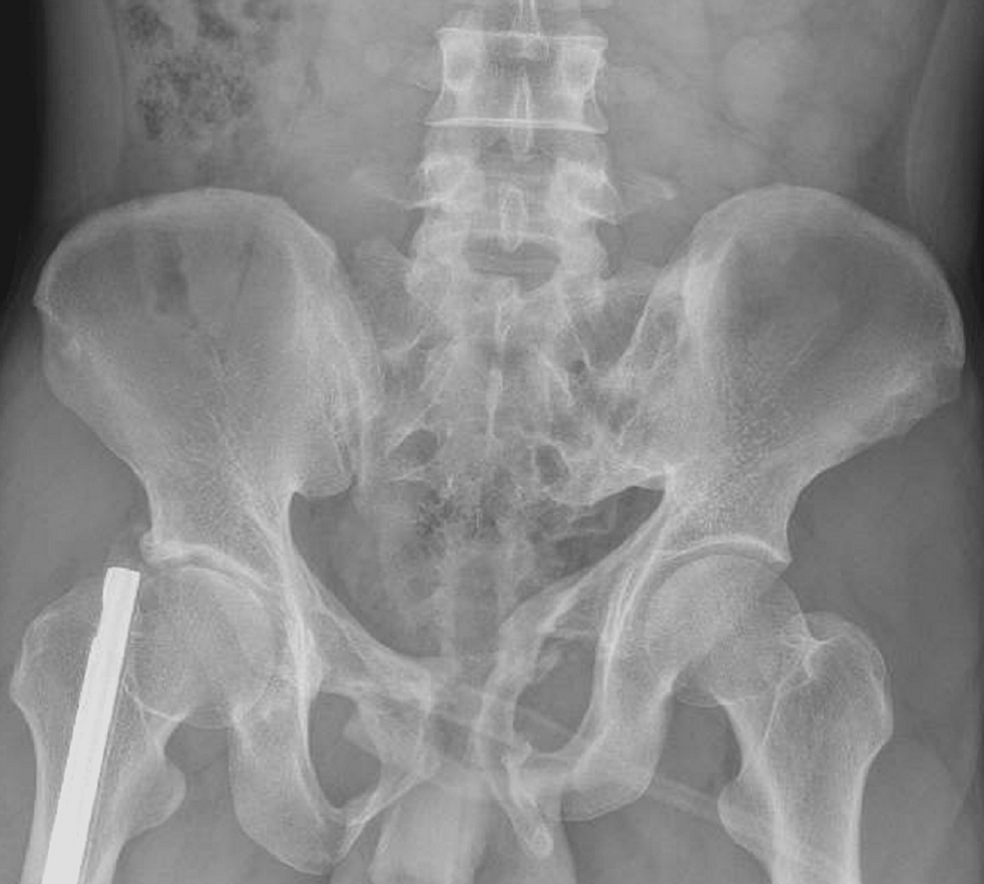
X-ray image of the pelvis The image shows the pelvic fractures and the right femur nail.

**Figure 2:**
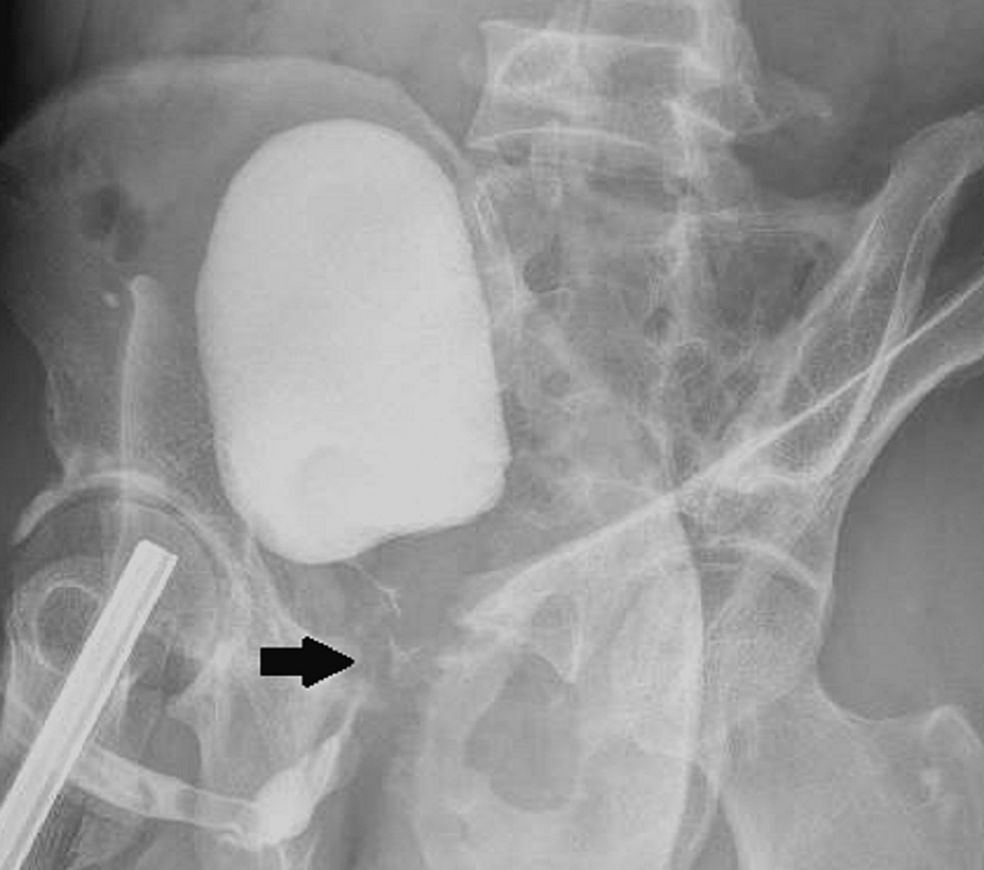
Retrograde urethrogram The arrow indicates the contrast defect suggesting the posterior urethral stricture with a length of 4 centimeters.

On physical examination upon admission, his vital signs were stable. He had old surgical scars in the mid-abdominal wall below his button belly and subscrotal line. The length of the penis was measured at 5 centimeters in the flaccid state. He had a normal penis and bilateral testicles on physical examination and 2D ultrasound. We are not qualified to do in-depth investigations to evaluate neurological or vascular causes such as penile duplex ultrasound and nocturnal penile tumescence. Urinalysis and midstream urine culture did not suggest urinary tract infection. The levels of sex hormones were within normal ranges (the level of serum testosterone was 16.76 nmol/L). In the uroflow parameters, the value of maximum flow rate (Qmax) was 4.4 ml/s, considered a bladder outlet obstruction. X-ray imaging studies revealed the previous lesions of pelvic fractures and the right femoral nail. On retrograde cystourethrogram, the length of posterior urethral stricture was estimated at 4 centimeters (Figures 1, 2).

**Figure 3:**
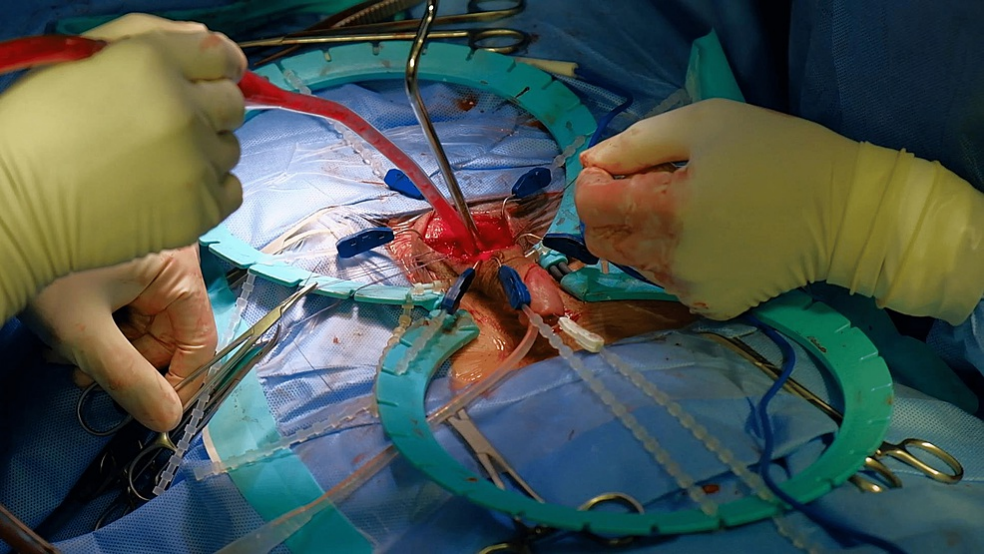
Corporal dilation Creating the cavity for the cylinders via dilation of the widespread fibrosis in bilateral corpora cavernosa

**Figure 4:**
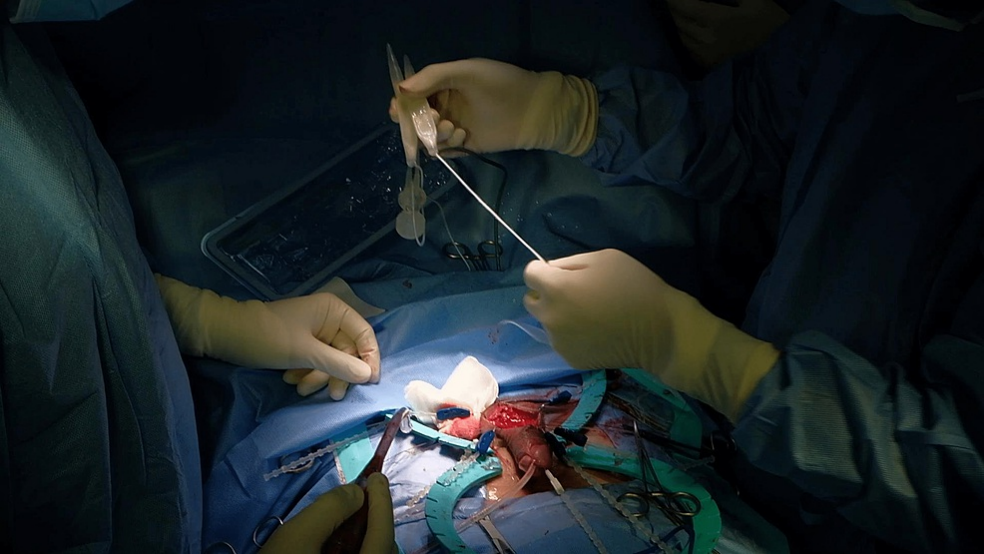
Cylinders preparation Cutting 0.5 centimeters of the hilts of two cylinders to fit inside the tunica albuginea

**Figure 5:**
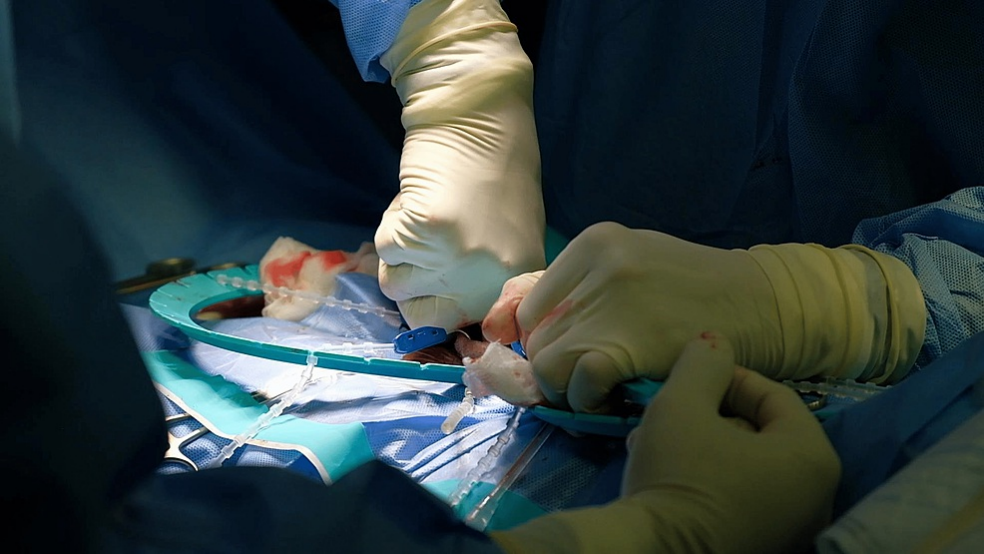
Reservoir placement Dissecting the left prevesical space for the reservoir with fingers

**Figure 6:**
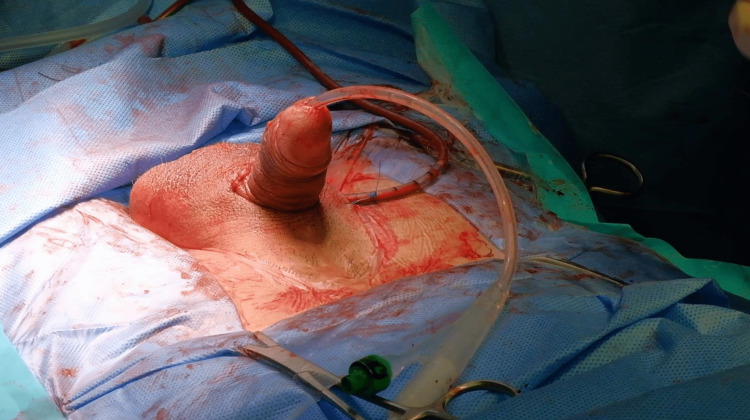
Devices testing Establishing a successful erection after surgery

Following diagnosis, we decided to perform urethral dilation under transurethral endoscopy and urethral catheterization with silicone Foley catheter 14 French. Seven days later, penile implant surgery was conducted with a penoscrotal incision. We utilized a three-piece inflatable prosthesis from Coloplast (The Titan® Penile Implants). During surgery, we recorded the extensive fibrosis of corpora cavernosa, particularly in the region near the posterior urethra, which hindered the process of corporal dilation. After dilation, we measured the length of the bilateral corporal cavernosa at 12 centimeters, meanwhile, the shortest cylinder length from the manufacturer was 11 centimeters. Thus, we had to shorten the hilts of two cylinders by 0.5 centimeters to ensure these cylinders fit in the patient’s cavernosa. We also chose the narrow base cylinders with small diameters. The other pitfall is reservoir placement in the left prevesical space due to the abdominal scar of a previous suprapubic cystostomy. We had to dissect this cavity gently with our fingers. After about two hours, we performed this procedure successfully (Figures 3-6) The postoperative progress was stable. We removed the drain and Foley catheter after 24 hours and five days, respectively. The patient was discharged after seven days.

In the sixth month of follow-up, the penile implant device still works well. The patient feels the urine stream is better. Notably, he could restore penile erection to achieve sexual intercourse and normal ejaculation. An evaluation of the IIEF-5 score was 20 at this time of follow-up. The intercourse satisfaction was significantly noted not only by the patient but also by his partner.

## Discussion

An estimated 3% of patients with ED were secondary after pelvic or perineal trauma [3]. Moreover, there has an intimate relationship between ED and urethra injury following a pelvic fracture. King et al. reported that impotence is seen in about 42% of patients with a urethral injury versus 5% of those without [4]. Chung et al. also found the incidence of ED in patients with PFUI (57%) was higher than in those with pelvic fractures alone [8]. Likewise, ED was more severe in PFUI patients. Munarriz et al. (1995) conducted a pharmacocavernosometry/pharmacocavernosography and pharmacoarteriography study in 131 men with persistent ED following blunt pelvic or perineal trauma. The results showed that cavernous artery insufficiency was found in 70% of patients, and corporeal veno-occlusive dysfunction was seen in 62% of those [9]. Shenfeld et al. studied 18/25 ED patients with posterior urethral stricture, underinvestigated nocturnal penile tumescence testing, and penile duplex ultrasounds in 41 PFUI patients. They found that 72% of cases were confirmed neurogenic defects and 28% of cases were related to arterial response on ultrasound findings [10]. Also, Fu et al. identified that 48% of patients were attributed to arterial ED, 15% had a venous leak, and 37% had non-vascular ED suggestive of neurogenic causes based on penile Doppler ultrasounds findings in 41 patients who suffered from pelvic fracture urethral distraction defects [11]. They proposed that the urethral damage, rather than being the direct cause of ED, serves as a stand-in for more extensive pelvic injury and increases the risk of neurovascular injury [8]. In this case, the patient had a long posterior urethral injury following a complex pelvic fracture, and additionally, he suffered from various urethral interventions, such as anastomotic urethroplasty, graft urethroplasty, and routine urethral dilation, which is a high risk for the development of de novo ED later. Although we could not perform the specialized tests to define the causation for ED, not only was the extensive corporal fibrosis attributed but also we theorized the contribution of neurovascular defects in ED. The scientists theorized that ED following urethroplasty could be caused by cavernous and/or perineal nerve injury and the disruption of antegrade blood flow through the corpus spongiosum [12]. However, two meta-analyses revealed that urethroplasty was not likely to cause de novo ED. Most ED cases were transient and reversed erectile function following reconstructive surgery at the long-term follow-up, which can last months or years after posterior urethral injury [13,14]. Thus, the long-term new-onset sexual dysfunction following pelvic trauma is probably caused by the injury itself rather than the reconstruction surgery.

**Figure 7:**
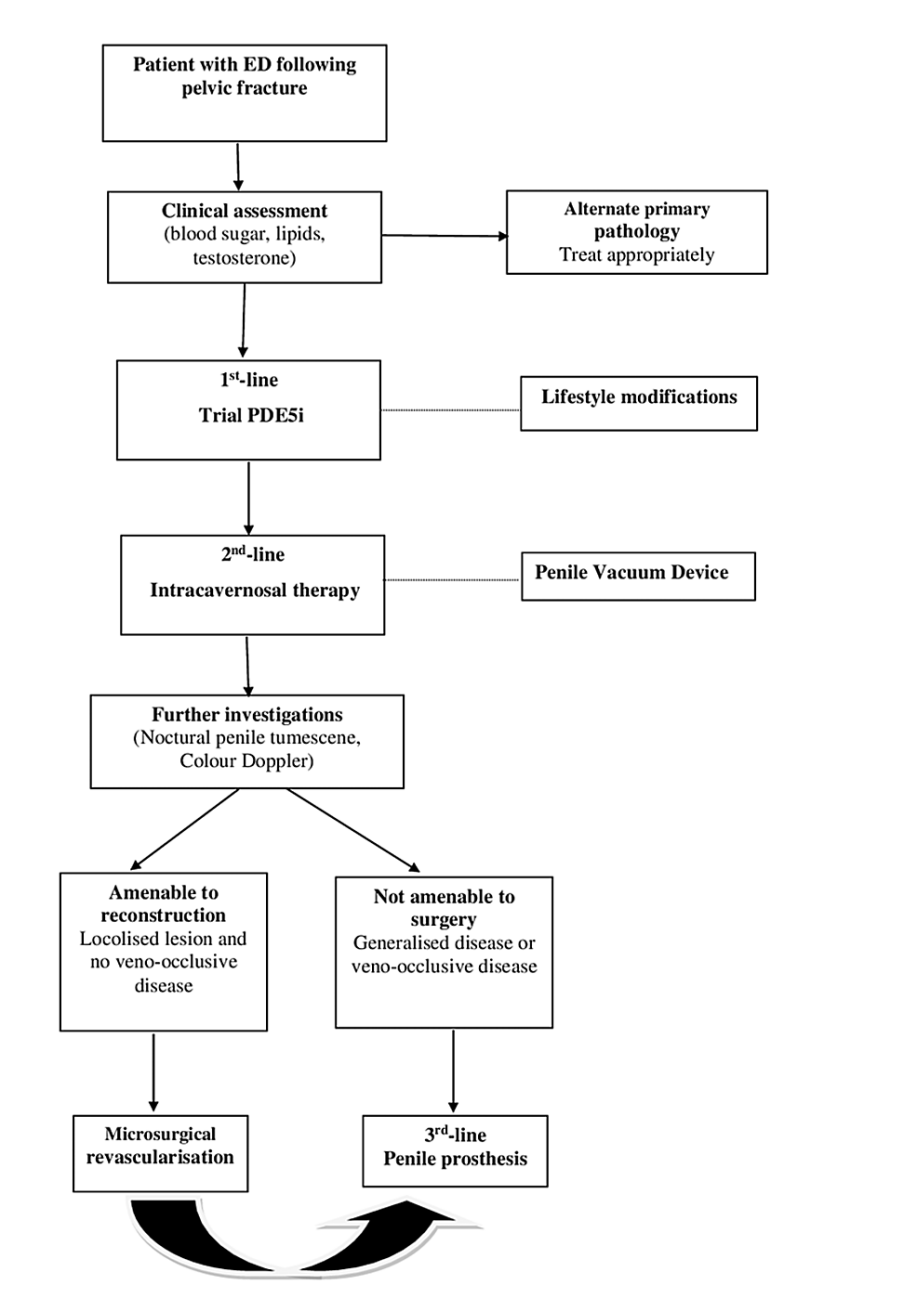
Algorithm for the management of ED patients following fracture of the pelvis Management of ED patients after a pelvic fracture includes three lines of treatments: oral PDE5i, intracavernosal injection, and penile prosthesis implantation, besides lifestyle changing and vacuum devices. Microsurgical revascularization was only indicated for a small few cases. Source: [3,5]

In regard to the treatment of ED in PFUI patients, PDE5i is the first-line treatment. Numerous pieces of preclinical and clinical evidence of cavernosal nerve injury reveal that PDE5i can prevent the fibrotic development of cavernosal tissue and enhance erectile function [15,16]. An intracavernosal injection is considered the second-line treatment that can then be tried if PDE5i is ineffective (Figure 7). There are several studies on medical treatments for the treatment of ED patients with PFUI. Shenfeld et al. (2004) evaluated the efficacy of PDE5i in patients with ED after posterior urethroplasty. The response rates were 60% and 20% in neurogenic and arteriogenic ED, respectively. Intracavernosal injection (ICI) therapy was used to treat patients who did not respond to sildenafil. Also, they found a higher improvement rate in the neurogenic ED group compared to the arteriogenic ED group (100% and 50%, respectively) [10]. Fu et al. (2012) reported that the overall success rate of sildenafil was approximately 81% [11]. Our patient was treated with oral PDE5i and used vacuum erectile devices for two years. However, the improvement response in erectile function was not noted. The explanation is likely due to the complex pathophysiology of ED in this case, which includes the combination of complete fibrosis in the corpora cavernosa and the complicated damage to the neurovascular system in that the long urethral stricture was surrogate.

Penile implant surgery has become the standard third-line treatment that is the definitive treatment for refractory ED cases of any etiology, including cases of pelvic trauma that have failed or refused more conservative therapies. Two modern designs of penile implant devices include semi-rigid (two-piece) and inflatable (three-piece) prostheses. Today, the inflatable penile prosthesis (IPP) is preferred and utilized in more than 80% of implant cases and is likely to be the gold standard, with the highest overall satisfaction rate of patients and partners being even more than 90% satisfaction as compared to other treatments. Nevertheless, this satisfaction rate can be lower in populations with extensive cavernous fibrosis and penile shortening due to traumatic injuries as well as urethral intervention procedures [17]. When we performed a penile implant procedure for our patient, we encountered several tough challenges. First, the extensive fibrosis of bilateral corpora cavernosa due to trauma and various urethral interventions impeded the dilation, which is the principal step in surgery. Besides, we utilized a penoscrotal incision that made it difficult to approach the corpora cavernosa. Second, in choosing two appropriate cylinders fitting inside the tunica albuginea, we did not use rear tip extenders as usual and had to shorten 0.5 centimeters to the two shortest cylinders from the manufacturer (11 centimeters) because, in clinical practice, we must choose a cylinder shorter by 1.5-2.0 centimeters than the corporal’s length, which aims to prevent from tubing erosion complications. The third challenge was creating the cavity for the reservoir with 60 ml of normal saline in the prevesical space because of fibrosis due to an old surgical scar in the abdominal wall and pelvic fracture. 

Like any medical implant, common complications, such as infection and malfunction, can be seen. The infection rate of penile prostheses was 0.5% to 3.5% and long-term device survival at 10 years ranged from 68% to 89% [15]. Currently, Chierigo et al. (2019) studied the long-term (≥ 15 years) complications of IPP. These findings showed that the device survival was 53% at the 20-year follow-up, and 49% experienced complications that included mechanical failure (79%), pain (12%), orgasmic dysfunctions (4.5%), or device infection (4.5%) [18]. In our case, the patient also has urethral stricture concomitantly and still may be at risk of urethral intervention later. Thus, the risk of infection and device failure may increase significantly, which requires close monitoring after surgery. Typically, to reduce the risk of prosthesis infection, the urethral stricture and pelvic fracture should be treated before penile implant placement. Few data reported the penile prosthesis implantation for ED following a pelvic fracture. Kardar et al. (2002) reported malleable penile prosthesis placement in an ED patient 19 months after the urethroplasty [19]. Eighteen months later, he developed a prosthesis infection and complete obliteration of the urethra. The penile prosthesis had to be removed and anastomotic urethroplasty was performed. The authors suggested utilizing an inflatable rather than a rigid one and reducing its length to minimize the prolonged stretching of the urethra and necrosis. Cui et al. also performed simultaneously urethroplasty and three-piece inflatable penile prosthesis re-implantation due to the twisted tubings that were palpated over the penile urethra [20]. Nevertheless, the risk of device infection was extremely high, so it is not routinely recommended. We have been very careful in preparing the patient before surgery, during the operation, and planning the postoperative follow-up. Before penile implant surgery, we had to examine urethral problems and urinary tract infections thoroughly. We conducted urethral dilation through endoscopy prior to prosthesis placement, as recommended, for two purposes. Initially, we treated the urethral stricture, which was quite complicated. Then, we put a urethral catheter before prosthesis implantation, which was a crucial step to avoid injuring the urethra when dilating the corpora cavernosa. During the implantation surgery, we ensured absolute sterility: operating room for transplantation, antibiotic-coated prosthesis devices, and continuous irrigation intraoperatively with a solution of vancomycin and gentamicin. We also utilized postoperative antibiotics, vancomycin and amikacin, for the high-risk patient for seven days. After the patient was discharged from the hospital, we planned to closely monitor the patient for urinary problems and the device working for a long time.

## Conclusions

Pelvic fracture is one of the common causes of ED. Penile implant surgery shows outstanding outcomes in ED unresponsive to medical therapy. However, it is necessary to diagnose and indicate as well as provide follow-up care after surgery in these ED patients with a urethral injury following a pelvic fracture due to the high risk of complications such as infection and device failure.
